# A Screening of a Library of T7 Phage-Displayed Peptide Identifies E2F-4 as an Etoposide-Binding Protein

**DOI:** 10.3390/molecules16054278

**Published:** 2011-05-24

**Authors:** Mihoko Takami, Yoichi Takakusagi, Kouji Kuramochi, Senko Tsukuda, Satoko Aoki, Kengo Morohashi, Keisuke Ohta, Susumu Kobayashi, Kengo Sakaguchi, Fumio Sugawara

**Affiliations:** Department of Applied Biological Science, Faculty of Science and Technology, Tokyo University of Science, 2641, Yamazaki, Noda, Chiba 278-8510, Japan

**Keywords:** etoposide (VP-16), T7 phage display, peptide, E2F-4, transcription, surface plasmon resonance

## Abstract

Etoposide (VP-16) is an anti-tumor compound that targets topoisomerase II (top II). In this study, we have identified an alternative binding protein of etoposide by screening a library of T7 phage-displayed peptides. After four rounds of selection using a biotinylated etoposide derivative immobilized on a streptavidin-coated plate, T7 phage particles that display a 16-mer peptide NSSASSRGNSSSNSVY (ETBP16) or a 10-mer NSLRKYSKLK (ETBP10) were enriched with the ratio of 40 or 11 out of the 69 clones, respectively. Binding of etoposide to these peptides was confirmed by surface plasmon resonance (SPR) analysis, which showed ETBP16 and ETBP10 to have a kinetic constant of 4.85 × 10^−5^ M or 6.45 × 10^−5^ M, respectively. ETBP16 displays similarity with the ser-rich domain in E2F-4, a transcription factor in cell cycle-regulated genes, suggesting that etoposide might interact with E2F-4 *via* this domain. SPR analysis confirmed the specific binding of etoposide to recombinant E2F-4 is in the order of 10^−5^ M. Furthermore, etoposide was shown to inhibit luciferase reporter gene expression mediated by the heterodimeric E2F-4/DP complex. Taken together, our results suggest that etoposide directly binds to E2F-4 and inhibits subsequent gene transcription mediated by heterodimeric E2F-4/DP complexes in the nucleus.

## 1. Introduction

**Figure 1:**
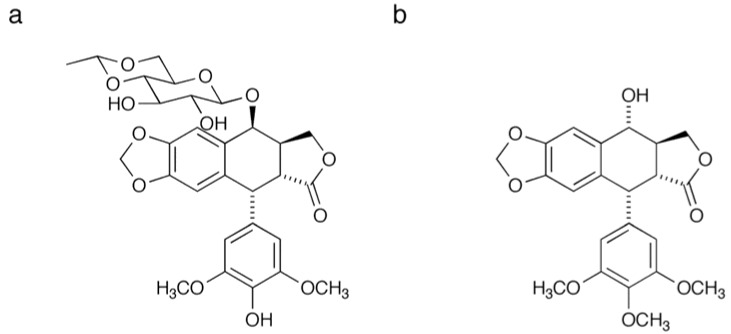
Structure of etoposide (**a**) and podophyllotoxin (**b**).

Etoposide (VP-16, Figure 1) is a semi-synthetic derivative of podophyllotoxin (Figure 1) that was originally isolated from the herbaceous perennial plant *Podophyllum peltatum* [1]. This compound stabilizes DNA-topoisomerase II (top II) cleavage complexes and arrests cell cycle in S or G2/M phase, which results in apoptotic cell death [2,3,4,5]. Etoposide displays a favorable therapeutic effect against specific solid cancers, such as those associated with the lung [3,6], bladder [7,8] and cervix [9,10,11], as well as leukemia [1,12]. Unfortunately, however, this agent also elicits serious side effects including myelosuppression, which limits the dosage range [2]. Understanding the molecular mechanism of action may contribute to not only explaining the therapeutic specificity, but also generating antagonistic concomitants or dosage schedules that could circumvent these serious toxicities. Thus, identification of direct binding target is imperative at the outset of these studies. There are several reports for biological or clinical observation of etoposide. These include induction of Smad6 in G1/S transition [13], G2 checkpoint activation *via* p38 MAP kinase [14], or importance of early G2/M checkpoint failure for etoposide-induced chromosomal aberrations [15]. However, little have been reported with regard to identification of the direct binding partner of etoposide other than top II.

**Figure 2:**
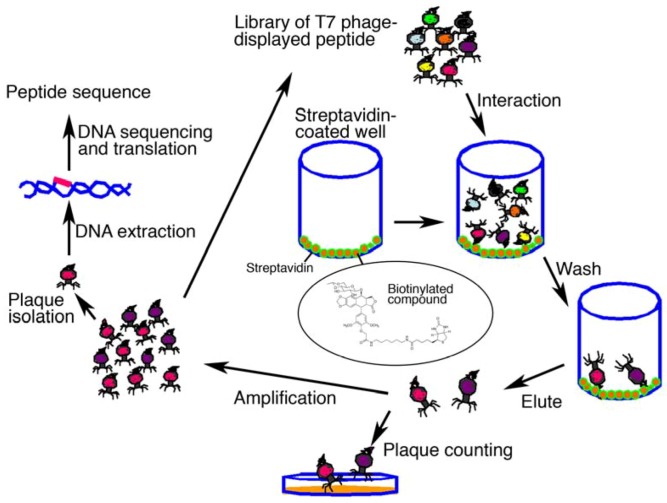
Schematic representation of affinity selection using phage display technology.

Phage display technology is a useful tool for the determination of the targets for small-molecule therapeutics (see ref. [16] and references therein). Gene engineering generates the phage library that displays foreign peptides on a coat protein. The library of phage-displayed peptides is screened using a bioactive small-molecule immobilized on a solid support. The small-molecule-recognizing sequence displayed on the phage can be efficiently determined by repeated rounds of selection (interaction, wash, elution and amplification using host bacteria) and sequencing of the relevant part of the phage DNA recovered from the eluate (Figure 2). Subsequent similarity search in the genome database using the resulting sequence enables prediction of the potential drug-binding species along with its binding site (see ref. [16] and references therein). Conventional proteomics approaches use soluble extracts from cells or tissues. By contrast, however, the phage display technique also facilitates the identification of less soluble proteins as drug-binding partners, such as transcription factors or membrane-associated receptors, by making use of similarity search between drug-selected peptides and these proteins. Furthermore, T7 phage-based screening procedures are superior to those of a filamentous phage-based system in some points; the rapid plaque formation properties (2–3 h) of T7 phage, the ability to directly infect even if the capsid is involved in drug binding (needs no elution conditions exploration) [16]. So far, use of this technique has allowed the successful identification of molecular target of various small-molecule therapeutics. These include anti-tumor, immunosuppressive, anti-diabetes, anti-lupus, anti-bacterial and anti-viral agents, and others (see ref. [16] and references therein).

In this study, we attempted an affinity selection using a synthetic biotinylated etoposide derivative immobilized on a 96-well streptavidin-coated microplate to identify etoposide-binding peptide (ETBP). Based on the resulting peptide sequence, we further evaluated the interaction between etoposide and synthetic ETBP or protein having similar sequence with the ETBP in detail. Based on the structural and functional information of the potential binding protein, we further elucidated the biological effects elicited by this interaction.

## 2. Results and Discussion

### 2.1. Synthesis of a Biotinylated Etoposide Derivative

**Scheme 1:**
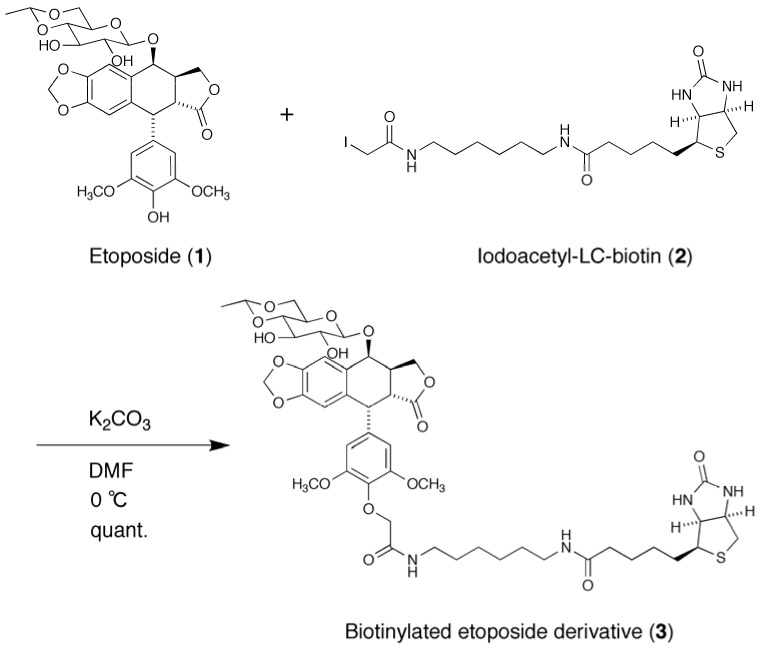
Synthesis of biotinylated etoposide derivative **3**.

To immobilize etoposide on a solid support, we employed the biotin-avidin system. A biotinylated etoposide derivative **3** was synthesized by a reaction of etoposide (**1**) and iodoacetyl-LC-biotin (**2**) under basic conditions as shown in Scheme 1. The solution of derivative **3** was added to a well of streptavidin-coated 96-well microplate and subjected to affinity selection. 

### 2.2. Affinity Selection of a Library of T7 Phage-displayed Peptides

**Figure 3:**
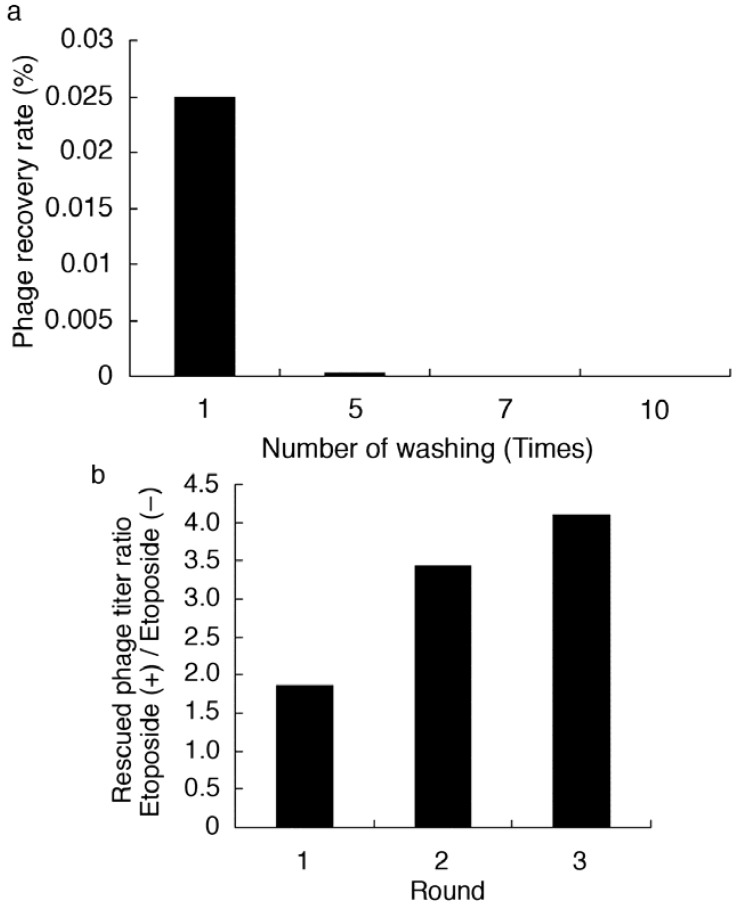
(**a**) Phage recovery rate in wash solution. Recovery rate (%) = titer of the washing fraction / titer of the input (1.0 × 10^8^ pfu) × 100. (**b**) Rescued phage titer in each round of selection.

**Figure 4:**
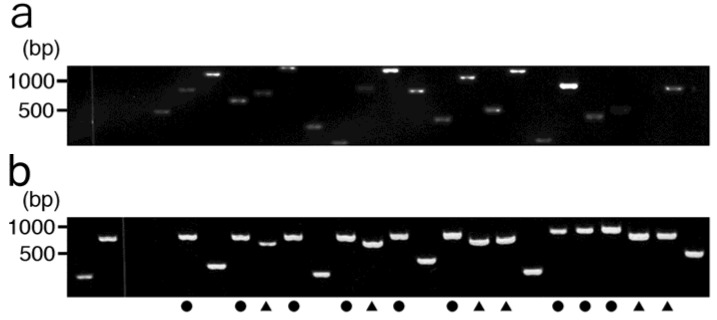
Agarose gel (1%) electrophoresis of recovered phage DNA. After each round of selection, part of phage DNA encoding the capsid protein from a set of arbitrarily selected phage particles (69 in total) were amplified by PCR and analyzed by electrophoresis. **(a)** After three rounds of selection. **(b)** After four rounds of selection. (●, ▲) Subsequent sequence analysis of the phage DNA showed that they encoded the peptides NSSASSRGNSSSNSVY (ETBP16, ●) or NSLRKYSKLK (ETBP10, ▲) as a fusion peptide of the capsid protein.

A library of T7 phage-displayed peptides was allowed to bind to the immobilized etoposide in order to screen for etoposide-binding T7 phage particles. According to preliminary tests of the selection procedure, appropriate conditions were determined and the total number of selection rounds were chosen. As a control, an etoposide-non-immobilized well was prepared and the titer of rescued phage was then compared to those from the etoposide-immobilized test wells. As shown in Figure 3a, remaining phage titer after washing five times was reduced by over 100-fold when using 1.0 × 10^8^ pfu of library, suggesting that unbound or non-specifically bound phages were efficiently removed by this procedure. The recovery ratio of the eluted solution after each round of selection was then determined (Table 1). As shown in Figure 3b, recovery ratio of phage particles gradually increased after each successive round of selection until the third round. However, no appropriate elution conditions were found for the fourth round of selection. Thus, bound phage DNA was directly recovered by host *E. coli* infection (BLT5615). Agarose gel electrophoresis of phage DNA recovered from selected phage in the elution solution of the third (a) and fourth (b) rounds of selection was carried out (Figure 4). As compared to the third round, enrichment of a specific band was clearly observed after the fourth round of selection. In particular, a PCR product with a size of 750 or 650 bp was most enriched (40 or 11 out of 69, respectively). Thus, the PCR product was subjected to base sequencing to determine the peptide sequence that potentially recognizes etoposide. As a result, a 16-mer NSSASSRGNSSSNSVY (ETBP16), and a 10-mer NSLRKYSKLK (ETBP10) sequence were determined as candidates of etoposide-recognizing peptides.

**Table 1:** Conditions for affinity selection using a biotinylated etoposide derivative and a streptavidin-coated 96-well microplate.

Immobil. etoposide		50 µL of 20 µM in 100 mM Tris-HCl (pH 8.0) − 2% DMSO soln. (4 °C, O/N)
Blocking		200 µL of 3% skimmed milk (r.t., 1 h)
Input of library		1.0 × 10^8^ pfu
Incubation		r.t., 3 h
Wash condition		200 µL of wash buffer for 5 times (5 min each on shaker)
Wash buffer		200 µL of 100 mM Tris-HCl (pH 8.0) containing 60 mM NaCl and 0.3% Tween 20
Elution condition		100 µL of elution buffer (O/N on shaker)
		100 µL of elution buffer for 4 times (5 min each on shaker)
		(Total 500 µL)
Elution buffer	Round 1	3 M NaCl
	2	A mixture containing 3 M NaCl, 2 M urea and 3% Tween 20
	3	A mixture containing 4 M NaCl, 2 M urea and 3% Tween 20
	4	Host *E. coli* (BLT5615) culture (100 µL for 30 min on shaker)

### 2.3. Interaction Analysis Between Etoposide and Synthetic ETBP Using SPR Biosensor

**Figure 5:**
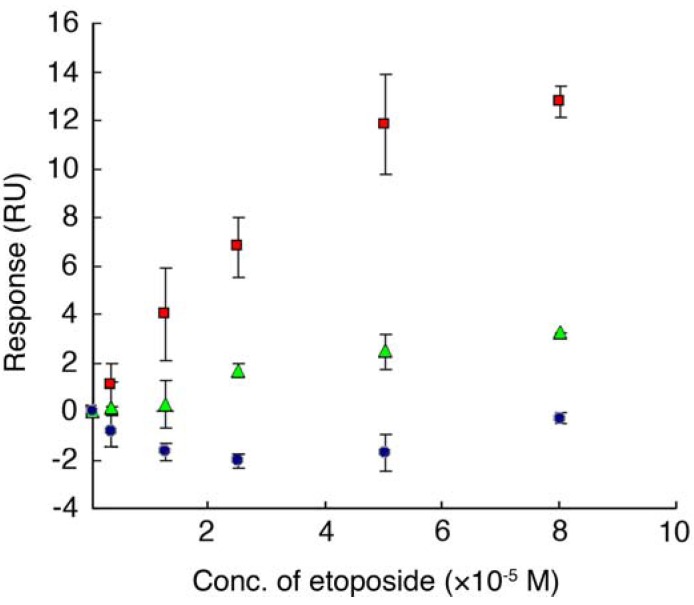
A plot of response (RU) obtained from SPR analysis between etoposide and synthetic peptide. Various concentrations of etoposide (3.2, 12.5, 25, 50 and 80 µM) were injected over the immobilized peptide on the sensor chip and the resulting response was detected. Plots were made by subtraction of the background signals generated simultaneously on the control flow cell (protein-non-immobilized cell), the injection of vehicle, and bulk response by DMSO. RU: resonance unit. 1 RU = 1 pg/mm^2^. 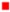, etoposide and ETBP16, 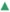, etoposide and ETBP10, 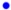, etoposide and a control peptide HG102. Data are means ± S.D. of at least three independent experiments.

Interaction between etoposide and ETBP16 was confirmed using surface plasmon resonance (SPR) biosensor, Biacore^®^3000 (GE Healthcare, Piscataway, NJ, USA) [17]. Synthetic ETBP16 or ETBP10 was immobilized on a CM5 censor chip by an amine coupling reaction. Various concentrations of etoposide were then injected over the peptide. As shown in Figure 5, a SPR response was detected upon injecting etoposide. These results suggest that both ETBP16 and ETBP10 interact with etoposide, as predicted from the initial phage display screening. By contrast, no response was observed against a control peptide HG102 (SGVMLGDPN). A global fitting using BIAevaluation 3.2 software (GE Healthcare) revealed that the dissociation constant between etoposide and ETBP16 or ETBP10 is 4.85 × 10^−5^ M and 6.45 × 10^−5^ M, respectively. 

### 2.4. Prediction of the Binding Partner for Etoposide by Similarity Search and Biological Data

A similarity search was performed using fasta3 and ETBP as a query. A large number of proteins that possess a segment of sequence similar to that of ETBP16 were found in the database (data not shown). It should be noted that similarity search guided by affinity-selected peptide (especially when the sequence is shorter) does not always result in identification of the proteins responsible for bioactivity of the small-molecules. Thus, biological data should be supportive and indispensable for dissecting the likely molecular target(s) from listed data.

**Figure 6:**
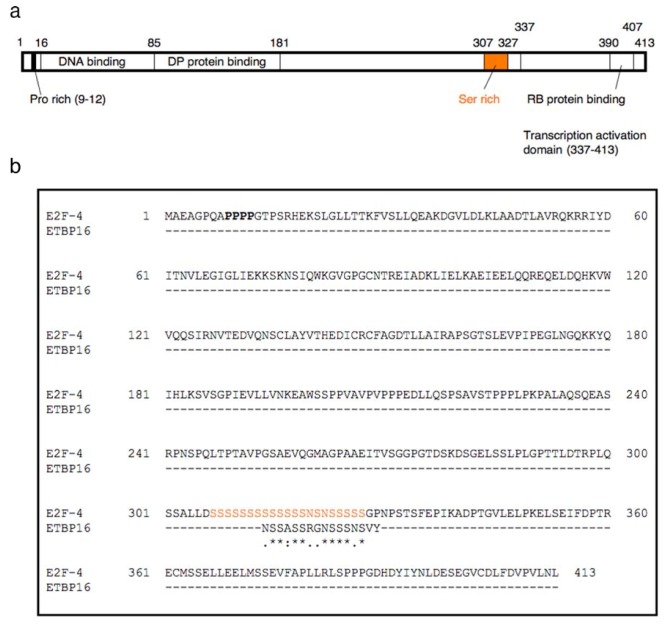
**(a)** Full length map of E2F-4. **(b)** Amino acid sequence of E2F-4 and similarity with ETBP16.

In the case of etoposide, many reports suggest that etoposide shows cell cycle delay as well as remarkable therapeutic effect for particular cancers, especially leukemia [1,12,18]. Based on these facts, we focused on E2F-4. E2F-4 is a transcription factor that forms a heterodimeric complex with DP protein and plays a central role in the expression of cell cycle-regulated genes [19,20,21]. The heterodimeric complex binds to the c/gGCGCg/c sequence of the consensus DNA-binding sites in the promoter region (GC box) *via* the DNA binding domain of the E2F-4 subunit (Figure 6a) and is capable of activating transcription [22], which leads to cell cycle progression. In particular, mutated E2F-4 appears to play an important role in malignant progression of particular cancers or leukemia [23,24,25]. Thus, interaction between etoposide and E2F-4 might contribute to reduced levels of transcription, resulting in a favorable chemotherapeutic effect for these malignancies.

ETBP16 shows similarity to the serine-rich domain (S307-S327) at the C-terminal end of E2F-4, which is distant from the DNA binding or DP protein binding domain located in the N-terminal region of the protein (Figure 6a and b). Next, we sought to validate this interaction and the subsequent biological effect in detail.

### 2.5. Interaction Analysis between Etoposide and E2F-4 Using an SPR Biosensor

**Figure 7:**
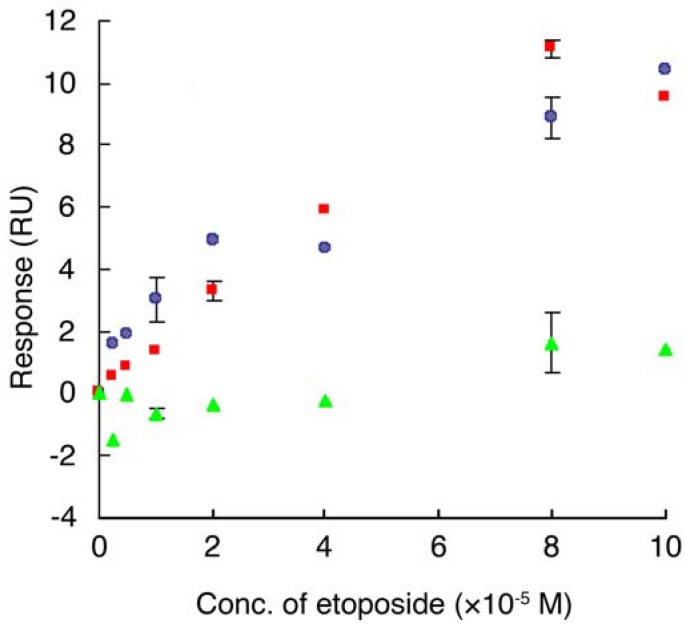
A plot of response (RU) obtained from SPR analysis between etoposide and protein. Various concentrations of etoposide (2.5, 5, 10, 20, 40, 80 and 100 µM) were injected over the immobilized protein on the sensor chip and the corresponding response was recorded. Plots were generated by subtraction of the background signals generated simultaneously on the control flow cell (protein-non-immobilized cell), the injection of vehicle, and bulk response by DMSO. RU: resonance unit. 1 RU = 1 pg/mm^2^.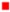, etoposide and GST-E2F-4, 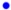, etoposide and top II, 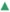, etoposide and GST. Data are means ± S.D. of at least three independent experiments.

Interaction between etoposide and E2F-4 was tested using an SPR biosensor. The gene encoding E2F-4 protein was engineered into an *E. coli* expression system as a GST fusion protein (GST-E2F-4) and purified by affinity interaction using glutathione Sepharose beads. The GST-E2F-4 was immobilized on a CM5 sensor chip using the same procedure as described for the synthetic ETBP. The chip was then tested to see whether binding with etoposide could be detected. A plot of SPR response as a result of the injection of a solution of etoposide at various concentrations is shown in Figure 7. We found that the SPR response increased upon injecting etoposide over the immobilized GST-E2F-4 in a dose-dependent manner. Similar results were obtained when GST-E2F-4 was replaced on the chip by top II, a known target of etoposide. The GST tag alone did not show any response in the concentration range of etoposide tested. Dissociation constant between etoposide and GST-E2F-4, or top II was determined to be 7.5 × 10^−5^ M and 4.9 × 10^−5^ M, respectively. These results suggest that etoposide associates with E2F-4.

### 2.5. Structure-binding Relationships of Etoposide for E2F-4

**Figure 8:**
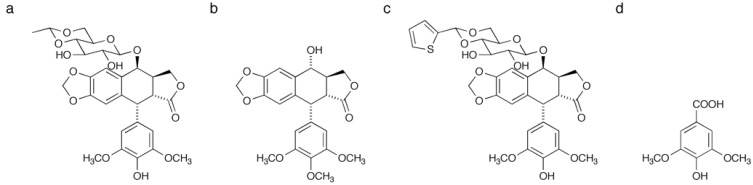
Structures of etoposide and its analogues used in this study: (**a**) etoposide; **(b**) podophyllotoxin; (**c**) teniposide; (**d**) syringic acid.

To ascertain the key structural features of etoposide for binding to E2F-4, we used etoposide analogues for SPR analysis (Figure 8). Among the three analogues tested, podophyllotoxin and teniposide were found to bind E2F-4, although the affinity was approximately four or nine-fold weaker than that of etoposide, respectively (Table 2). Syringic acid, which corresponds to a partial structure of etoposide, showed no response in the concentration range tested. Taken together, these results suggest that the glucose moiety in etoposide is important for binding to E2F-4. Moreover, replacement of the methyl moiety with a thiophene group appears to interfere with E2F-4 binding.

**Table 2:** Dissociation constants between GST-E2F-4 and each compound.

Compound:	Etoposide	Podophyllotoxin	Teniposide	Syringic acid
K_D_ (×10^−5^ M):	7.5	30.8	65.4	ND

### 2.6. Luciferase Reporter Gene Assay

**Figure 9:**
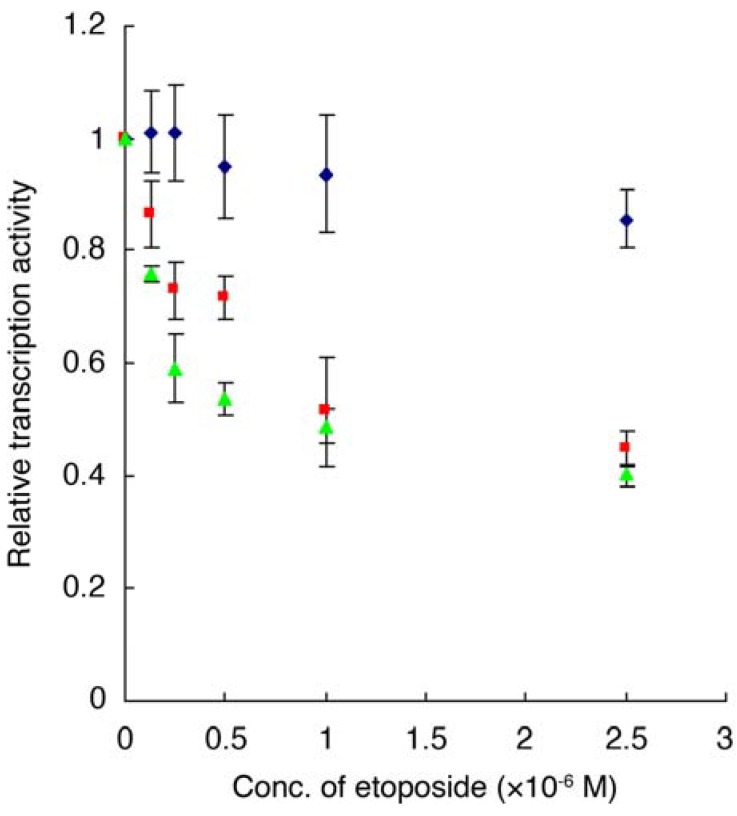
Relative luminescence in the presence or absence of etoposide. 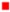, E2F4/DP1 expressing cell, 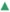, E2F-4/DP2 expressing cell, 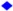 , control cell transfected with vehicle. Data are means ± S.D. of three independent experiments.

A luciferase reporter gene assay was performed in order to elucidate the biological effect triggered by etoposide binding to E2F-4. E2F-4 functions as a transcription factor, forming a heterodimeric complex with DP *via* the DP binding domain (G86-I181) (Figure 9) [22]. The E2F-4 and DP genes were co-transfected in CHO-K1 cells. E2F-4/DP complex-dependent expression of a reporter gene (*Cypridina* luciferase) in the GC box downstream was then detected in the presence or absence of etoposide.

Various concentrations of etoposide, which show no effect in terms of cell growth in a MTS assay (data not shown), were incubated for 15 h with the cells and any effect on transcription mediated by the E2F-4/DP complex was monitored. Luciferase-mediated luminescence, which is dependent on luciferase gene transcription, decreased in the presence of etoposide in a dose-dependent manner using E2F-4/DP1 expressing cell lines. This result indicates that etoposide inhibits the reporter gene transcription mediated by each complex. The IC_50_ of transcription by E2F-4/DP1 was 1.56 µM. In addition, etoposide also inhibited reporter gene transcription mediate by E2F1/DP1 complex (IC_50_: 3.59 µM, Table 3). These are relatively low concentrations than K_D_ value between etoposide and E2F-4 (75 µM, Table 2) or top II (49 µM), which were obtained from the SPR analysis, or even the top II inhibitory concentration *in vitro* (62 µM) [5]. We predict that, in the cells, etoposide might show stronger affinity with the targets compared to *in vitro* experiment conditions. Alternatively, there may be portions of DP protein that is sterically close to Ser-rich domain in E2F-4 and is involved in the etoposide binding. Furthermore, dual or multiple intracellular actions of etoposide might strengthen its biological effects.

**Table 3:** IC_50_ of reporter gene transcription of etoposide.

Complex:	E2F-4/DP1	E2F-4/DP2	E2F-1/DP1
IC_50_ (µM) of etoposide	1.56	1.16	3.59

It is well explained that etoposide inhibits top II and stabilizes double strand breaks (DSBs), which elicits G2/M arrest and cell cycle delay, and results in apoptotic cell death [4,26]. Furthermore, it has been reported that E2F/DP complex induces G1 to S phase transition and accumulates G1 phase cells by inhibition of the E2F/DP transcription [13,19]. It is also known that G1 to S phase transition is also observed by the treatment of etoposide [13]. However, in our experiment, etoposide rather inhibited reporter gene expression mediated by this complex. This apparent discrepancy might indicate that top II inhibition of etoposide is the main mechanism for cell cycle delay or apoptosis and binding of etoposide to E2F contributes little to the cell cycle regulation of this compound. Nevertheless, specific cancers or leukemia in which E2F-4 is mutated and constitutively activated seem to be sensitive for the treatment of etoposide *in vivo* [12,25]. Binding of etoposide to E2F, which has been identified in this study, might explain such favorable therapeutic effect or specificity. Further studies of these mechanisms, including binding of etoposide to other E2F subtypes and its effect on the formation of heteromeric complexs, are currently underway.

## 3. Experimental

### 3.1. Instrumentation

^1^H- and ^13^C-NMR data were obtained from a Bruker 600 MHz spectrometer (Avance DRX-600) or a 400 MHz spectrometer (Avance DRX-400), respectively. Mass spectral data was collected on an ABI QSTAR [Applied Biosystems Japan (ABI), Tokyo, Japan]. PCR was performed using a PTC-200 (Peltier Thermal Cycler; Bio-Rad, Hercules, CA, USA). Sequencing analysis was carried out using an ABI PRISM 3100 genetic analyzer (Applied Biosystems, Foster City, CA, USA) with BigDye^®^ Terminator v3.1 Cycle Sequencing Kit (Applied Biosystems). Centrifugation was performed using a Centrifuge 5417R (Eppendorf, Tokyo, Japan). Fmoc peptide synthesis was carried out using a peptide synthesizer PS-3 (Aloka, Tokyo, Japan). Peptide purification was performed using a reverse phase preparative high performance liquid chromatography (HPLC) instrument (SSC-3461, Senshu Scientific, Tokyo, Japan) with a CAPCELL PAK C-18 column (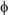
20 × 250 mm, UG120Å, Shiseido, Tokyo, Japan). Synthetic peptide was verified by a LC-MS using Bruker Daltonics Esquire 3000 plus (Bruker Daltonics K. K., Kanagawa, Japan). Recombinant GST-E2F-4 was purified using a fast performance liquid chromatography (FPLC) instrument (ÄKTA explorer 10s, GE Healthcare, Piscataway, NJ, USA) with 1 mL of GSTrap column (GE Healthcare). The SPR biosensor (Biacore^®^3000), CM5 sensor chip and an amine coupling kit were from GE Healthcare. Optical density (OD) was determined using Wallac Victor^TM^ 1420 multilabel counter (PerkinElmer, Waltham, MA, USA).

### 3.2. Materials

#### 3.2.1. Chemistry

Etoposide was obtained from SIGMA-Aldrich (St. Louis, MO, USA). Iodoacetyl-LC-biotin was purchased from Pierce (Rockford, IL, USA). Fmoc amino acid was from Calbiochem (Darmstadt, Germany). Special grade of organic solvents were used unless otherwise noted. 

#### 3.2.2. Biology

T7select10-3 OrientExpress^TM^ cDNA Cloning System and pET28a(+) were purchased from Novagen (Madison, WI, USA). Restriction enzyme was purchased from Takara Bio Inc. (Shiga, Japan). A mouse anti-GST antibody and an anti-mouse IgG antibody AP conjugate were from SIGMA-Aldrich. Ham’s F-12 medium was purchased from Nacalai Tesque (Kyoto, Japan) Fetal calf serum was from Thermo Trace (Victoria, Australia). Lipofectamine 2000 and Opti MEM medium were obtained from Invitrogen. MTS assay was performed using CellTiter 96^®^ Aqueous Non-Radioactive Cell Proliferation Assay kit (Promega, Madison, WI, USA). Luciferase reporter gene assay was performed using Dual-Luciferase Reporter Assay System (Promega). pGL3 vector was from Promega.

### 3.3. Synthesis of Biotinylated Etoposide Derivative

To a solution of etoposide (**1**, 10.6 mg, 18.0 µmol) and *N*-iodoacetyl-*N*-biotinylhexylenediamine (**2**, 9.5 mg, 18.6 µmol) in DMF (2.0 mL) was added K_2_CO_3_ (4.0 mg, 28.9 µmol) and the mixture was stirred at 0 °C for 9 h (Scheme 1). Solvents were removed under a reduced pressure. The residue was purified by silica gel chromatography (CHCl_3_-MeOH = 9:1) to yield etoposide-biotin conjugate (15 mg, quant.). [α]_D_^25^ = −18.5 (*c* 0.50, MeOH); ^1^H-NMR (600 MHz, CDCl_3_-CD_3_OD) δ 7.87 (1H, m), 6.85 (1H, s), 6.61 (1H, m), 6.26 (2H, s), 6.01 (1H, s), 5.98 (1H, s), 4.98 (1H, d, *J* = 3.2 Hz), 4.76 (1H, q, *J* = 4.9 Hz), 4.73 (1H, s), 4.62 (1H, d, *J* = 5.4 Hz), 4.56 (1H, d, *J* = 7.7 Hz) 4.50 (2H, m), 4.47 (2H, *brs*), 4.31 (2H, m), 4.19 (1H, dd, *J* = 10.7 Hz, 4.2 Hz), 3.73 (6H, s), 3.67 (3H, m), 3.59 (1H, m), 3.55 (1H, t, *J* = 7.3 Hz), 3.49 (1H, dd, *J* = 14.0 Hz, 5.4 Hz), 3.33 (2H, m), 3.28–3.14 (3H, m), 2.93–2.86 (4H, m), 2.70 (1H, d, *J* = 12.7 Hz), 2.19 (2H, m), 1.68 (4H, m), 1.58–1.42 (2H, m), 1.37 (3H, d, *J* = 4.9 Hz); ^13^C-NMR (100 MHz, CDCl_3_-CD_3_OD) δ 175.6, 173.5, 170.3, 163.8, 152.3, 152.3, 148.7, 147.0, 136.0, 135.7, 132.5, 127.8, 110.6, 109.4, 107.9, 101.6, 101.1, 99.7, 79.9, 74.3, 72.5, 68.0, 67.8, 66.2, 61.7, 60.0, 56.0, 56.0, 55.4, 43.8, 40.9, 40.3, 39.9, 39.1, 39.0, 37.7, 35.8, 29.1, 28.2, 27.9, 26.2, 25.6, 25.9, 25.5, 20.1, 18.0; IR (film) cm^−1^ 3354, 3018, 2934, 1736, 1699, 1592, 1506, 1484, 1459, 1419, 1334, 1216, 1160, 1126, 1039, 931, 760, 668; HRMS (ESI) calcd for C_47_H_62_N_4_O_16_SNa ([M+Na]^+^) 993.3773, found 993.3767.

### 3.4. Screening of a Library of T7 Phage-displayed Peptide

The key selection conditions are summarized in Table 1. Biotinylated etoposide derivative (**3**) was immobilized on a streptavidin-coated 96-well microplate at 4 °C, O/N. The wells were then blocked with 3% skimmed milk in 100 mM Tris-HCl (pH 8.0). An aliquot (1.0 × 10^8^ pfu) of the library of T7 phage was allowed to bind to the etoposide for 3 h. After washing five times with 200 µL of Tris-HCl (pH 8.0) containing 60 mM NaCl and 0.3% Tween 20 to remove non-specifically bound phage, the remaining phage particles were rescued using an appropriate elution buffer in each round as shown in Table 1. Following three or four rounds of selection, 69 plaques were randomly picked from LB plates and each was dissolved in phage extraction buffer (100 mM NaCl, 6 mM MgSO_4_ in 20 mM Tris-HCl, pH 8.0). The phage DNA was then analyzed by agarose gel electrophoresis and subsequently sequenced. Plaque formation or counting was performed according to the manufacture’s instructions.

### 3.5. Agarose Gel Electrophoresis and DNA Sequencing

Agarose gel electrophoresis and DNA sequencing were performed as described previously [27].

### 3.6. Synthesis and Purification of Peptide

The proposed etoposide-recognizing peptide (ETBP16 and ETBP10) was synthesized by the Fmoc method using a peptide synthesizer PS-3 (Aloka) according to previous reports [28]. The synthetic product was purified using a reverse phase preparative HPLC instrument (SSC-3461, Senshu Scientific) equipped with a CAPCELL PAK C-18 column (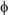
20 × 250 nm, UG120Å, Shiseido) and verified by LC-MS using the Bruker Daltonics Esquire 3000 plus.

### 3.7. Protein Expression and Purification

The 1239-bp cDNA fragment of E2F-4 was amplified from I.M.A.G.E cDNA clone (clone ID CL0B005ZB12, Invitrogen) by PCR with the primers 5’-GGATCCATGGCGGAGGCCGGG-3’ (up) and 5’-CTCGAGTCAGAGGTTGAGAACAGGCAC-3’ (down) and subcloned into pGEM-T vector (Promega). Following purification and digestion with *Eco*RI and *Xho*I, the cDNA fragment was subcloned into the expression vector pET28a(+) (Novagen). The construct was transformed into *E. coli* BL21(DE3)pLysS (Novagen). Heterologous gene expression was induced by addition of 0.1 mM isopropyl β-D-1-thiogaractopyranoside (IPTG). The culture was continued at 37 °C with vigorous shaking for 1 h. The cells were then harvested and suspended in binding buffer (0.5 M NaCl, 5 mM 2-mercaptoethanol in 50 mM sodium phosphate, pH 7.5). Following sonication and centrifugation the soluble fraction was obtained and purified using the FPLC instrument and GSTrap column (1 mL, GE Healthcare) and then subjected to kinetic analysis by SPR. The purified GST-E2F-4 in resulting fraction was detected by a SDS-PAGE followed by CBB staining or Western blotting using rabbit anti-GST (primary) and anti-rabbit IgG-AP conjugate (secondary) antibodies (Sigma-Aldrich) with BCIP/NBT solution.

### 3.8. Interaction Analysis Using an SPR Biosensor

Analysis of binding between etopside and synthetic ETBP or recombinant GST-E2F-4 was performed with a surface plasmon resonance (SPR) biosensor (Biacore^®^3000, GE Healthcare) [17]. The synthetic ETBP or protein dissolved in each buffer (ETBP16: 10 mM acetate buffer, pH 4.0−10% DMSO; ETBP10, HG102: 10 mM carbonate buffer, pH 8.5; protein: 10 mM acetate buffer, pH 4.0) was injected over a CM5 sensor chip at 10 µL/min and captured on the carboxymethyl dextran matrix *via* an amine coupling reaction. The surface was activated by injecting a solution containing 200 mM EDC and 50 mM NHS for 14 min. The protein or peptide was injected and the surface was then blocked by injecting 1 M ethanolamine at pH 8.5 for 14 min. This reaction immobilized 1279 resonance units (RU) of ETBP16, 3400 RU of ETBP10, 350 RU of HG102, 11421 RU of GST-E2F-4, 4388 RU of GST, and 7500 RU of top II (usb). Binding analysis with etoposide (six different concentrations) was performed in PBS buffer with 10% DMSO using a flow rate of 20 µL/min at 25 °C. BIAevaluation 3.2 software (GE Healthcare) was used to determine the kinetic parameters.

### 3.9. Cell Culture

CHO-K1 cells were cultured in Ham’s F-12 (Nacalai Tesque) supplemented with 10% fetal calf serum (Thermo Trace). The cells were cultured in 75 cm^2^ BD Falcon^TM^ cell culture flasks (BD Biosciences, San Jose, CA) at 37 °C in a humidified chamber containing 5% CO_2_. The culture medium was changed every second day.

### 3.10. MTS Assay

Cell proliferation was evaluated using a colorimetric [3-(4,5-dimethylthazol-2-yl)-5-(3-carboxy-methoxyphenyl)-2-(4-sulfophenyl)-2H-tetrazolium, inner salt] (MTS) assay (Promega). The cells (2 × 10^4^ cells) were plated in 96-well plates and incubated at 37 °C. Once the cells were 90% confluent, a solution of etoposide dissolved in 0.1% DMSO-Opti MEM medium (Invitrogen) at various different concentrations was added into the well (final 200 µL/well). The cells were further incubated for 24 h at 37 °C. A 40 µL aliquot of MTS reagent was added to each test well followed by an additional incubation at 37 °C for 60 min. Cell proliferation was determined by measuring optical density at 490 nm.

### 3.11. Transfection

The vectors (pCMV-E2F-4, pCMV-DP1, pCMV-DP2) for the luciferase reporter gene assay were kindly provided by Dr. Junji Magae (Institute of Research and Innovation (IRI), Kashiwa, Chiba, Japan) The reporter gene plasmid (pGL3-GC box) was constructed by insertion of double stranded DNA with *Xho*I and *Hind*III site, which was generated by an annealing of 60 pmol of oligonucleotides 5’-TCGAGTTTCGCGCTTTCGCGCTTTCGCGCTTTCGCGCTTTCGCGCTTTCGCGCA-3’ and 5’-AGCTTGCGCGAAAGCGCGAAAGCGCGAAAGCGCGAAAGCGCGAAAGCGCGAAAC-3’ into pGL3 vector (Promega). Transfection was then performed using Lipofectamine 2000 (Invitrogen) in a 96-well format according to the manufacturer’s instructions (Invitrogen). 

### 3.12. Luciferase Reporter Gene Assay

The Dual-Luciferase Reporter Assay System (Promega) was used to test whether etoposide inhibits transcription mediated by the heterodimeric E2F-4/DP complex. Six hours after transfection with the E2F-4 and DP genes, CHO-K1 cells were incubated with various concentrations of etoposide dissolved in 0.1% DMSO-Opti MEM medium (Invitrogen, 100 µL/well). After incubation at 37 °C for 15 h and washing twice with PBS, cells were lysed by adding 25 µL of Passive Lysis buffer (Promega) and further mixed with 45 µL of Luciferase Assay buffer (Promega) for luminescence reactions using *Cypridina* luciferin. Luminescence intensity was determined by measuring optical density at 488 nm. Stop-Glo buffer (Promega) was then added to repress the *Cypridina* luciferin luminescence and simultaneously enhance the luminescence by *Renilla* luciferin. The optical density at 488 nm was measured and used as a background correction.

### 3.13. Bioinformatics Tool

In order to search the proteins including the etoposide-binding sequence, we screened a large number of proteins registered in the Human Genome Database. As a convenience, fasta3 program (http://www.ebi.ac.uk/fasta33/), Swiss-Prot new library (version 3.4t21) and the expert protein analysis system (ExPASy) were utilized for the scanning.

## 4. Conclusions

In the present study, we identified NSSASSRGNSSSNSVY (ETBP16) and NSLRKYSKLK (ETBP10) as etoposide-recognizing peptides by screening a library of T7 phage-displayed peptides. Interaction analysis using SPR confirmed the etoposide binding to these peptides with a dissociation constant in the order of 10^−5^ M. Subsequent similarity search and SPR analysis confirmed the etoposide-binding protein to be E2F-4, a transcription factor that plays a central role in the expression of cell cycle-regulated genes. The likely binding site was predicted to be a ser-rich domain in the C-terminal region of E2F-4. Among the four etoposide analogues tested in this study, the affinity between etoposide and E2F-4 was the strongest. Furthermore, a reporter gene assay showed that etoposide inhibits gene transcription mediated by the heterodimeric E2F-4/DP complex. Overall, it is concluded that etoposide directly and specifically binds to E2F-4 *via* the ser-rich domain, resulting in inhibition of the downstream gene transcription. We believe the data obtained in this study will contribute to not only understanding the molecular mechanism of action or therapeutic specificity for etoposide *in vivo*, but also generating superior derivatives with reduced side effects by drug design.
